# Neural effects of muscle stretching on the spinal reflexes in multiple lower-limb muscles

**DOI:** 10.1371/journal.pone.0180275

**Published:** 2017-06-29

**Authors:** Yohei Masugi, Hiroki Obata, Daisuke Inoue, Noritaka Kawashima, Kimitaka Nakazawa

**Affiliations:** 1 Department of Life Sciences, Graduate School of Arts and Sciences, The University of Tokyo, Komaba, Meguro-ku, Tokyo, Japan; 2 Department of Rehabilitation for the Movement Functions, Research Institute of the National Rehabilitation Center for Persons with Disabilities, Namiki, Tokorozawa-shi, Saitama, Japan; Tokai University, JAPAN

## Abstract

While previous studies have shown that muscle stretching suppresses monosynaptic spinal reflex excitability in stretched muscles, its effects on non-stretched muscles is still largely unknown. The purpose of this study was to examine the effects of muscle stretching on monosynaptic spinal reflex in non-stretched muscles. Ten healthy male subjects participated in this study. Muscle stretching of the right triceps surae muscle was performed using a motor torque device for 1 minute. Three different dorsiflexion torques (at approximately 5, 10, and 15 Nm) were applied during muscle stretching. Spinal reflexes evoked by transcutaneous spinal cord stimulation were recorded in both the lower-limb muscles before, during, and at 0 and 5 min following muscle stretching. The amplitudes of the spinal reflexes in both the stretched and non-stretched muscles in the right (ipsilateral) leg were smaller during stretching compared to before, and at 0 and 5 min after stretching. Furthermore, the degree of reduction in the amplitude of the spinal reflexes in the right (ipsilateral) leg muscles increased significantly as the dorsiflexion torque (i.e., stretching of the right triceps surae muscles) increased. In contrast, reduction in the amplitude of the spinal reflexes with increasing dorsiflexion torque was not seen in the left (contralateral) leg muscles. Our results clearly indicate that muscle stretching has inhibitory effects on monosynaptic spinal reflexes, not only in stretched muscles, but also in non-stretched muscles of the ipsilateral leg.

## Introduction

Static muscle stretching is widely used to enhance muscle flexibility in the fields of sport and rehabilitation. Recent studies have reported that in addition to mechanical factors [1, 2], the effects of muscle stretching may occur due to neural factors [3–7]. For example, spinal reflex excitability, as assessed using the Hoffmann (H)-reflex, was suppressed in the soleus (Sol) muscle during [3–7] and up to 2 min following [8] triceps surae muscle stretching. While the amplitude of the direct motor response (muscle action potential, M-wave) was not affected by muscle stretching, the degree of suppression of Sol muscle H-reflexes increased when the dorsiflexion angle was increased [6]. These results indicate that muscle stretching has inhibitory effects on monosynaptic spinal reflex excitability in stretched muscles. One of the mechanisms suggested to underlie this inhibitory effect is afferent inputs from intramuscular receptors (such as the muscle spindle and the Golgi tendon organ), which can inhibit spinal reflex excitability [1, 7, 9]. However, the neural effects of muscle stretching are not fully understood. For example, it is unknown whether muscle stretching in one leg modulates monosynaptic spinal reflex excitability in the non-stretched muscles of the ipsilateral leg, and whether muscle stretching in one leg modulates the spinal reflex excitability in the contralateral leg.

In the previous study, the non-local effects of muscle stretching have been documented with increased flexibility of the contralateral hamstrings following unilateral hamstrings stretching [10], as well as increased flexibility of the upper body musculature (shoulder) after stretching of the lower body (hips), and vice versa (increased lower body flexibility following stretching of the upper body) [11]. The authors attributed the increases in range of motion (ROM) for the non-local joint to stretch-induced neural inhibition, as the increased non-local ROM cannot be attributed to increases in muscle compliance or viscoelastic tissue changes [11]. However, there is no direct evidence that muscle stretching has neural effects on non-stretched muscles. Neurophysiological studies have demonstrated evidence for neural connections between afferent fibers from the local muscle and motoneurons innervating non-local muscles. For example, the direct projections of muscle spindle Ia afferents to the motoneurons of various leg muscles (so-called “heteronymous Ia connection”) were shown in animal [12] and human studies [13, 14]. In addition, groups Ⅰ and Ⅱ afferent fibers of contralateral leg muscles also affect the reflex excitability by means of interneurons interposed in the crossed pathway [15–21]. Considering these results, it is possible that muscle stretching would have neural effects on the excitability of monosynaptic spinal reflex, not only in stretched muscles, but also in non-stretched muscles.

H-reflex testing can be used to assess spinal reflex excitability in only a few muscles, such as the Sol muscles; however, it cannot be used to test multiple lower-limb muscles in both legs simultaneously [22]. The recently developed transcutaneous spinal cord stimulation (tSCS) can evoke spinal reflexes in multiple lower-limb muscles in both legs simultaneously [23–25]. The spinal reflex elicited by tSCS is thought to mainly reflect the excitability of the monosynaptic spinal reflex arc [23]. Using tSCS, we can evaluate the neural effects of muscle stretching on spinal reflex excitability in multiple lower-limb muscles. Therefore, the purpose of this study was to investigate the neural effects of muscle stretching on non-stretched muscles. To this end, we investigated spinal reflex excitability in bilateral lower-limb muscles using the tSCS technique before, during, and after stretching of the unilateral triceps surae muscles.

## Materials and methods

### Participants

This study was conducted in accordance with the Declaration of Helsinki (1964) and with the approval of the Human Ethics Committee of the National Rehabilitation Center for Persons with Disabilities (NRCD, Saitama, Japan). Ten healthy male subjects [mean age: 27.9 ± 6.4 (SD) years] participated in this study. None of the subjects had any known history of neurological impairments and trained regularly with sports teams or clubs. Each subject provided written informed consent to participate in the experimental procedures.

### Experimental setup and protocol

**Fig 1:**
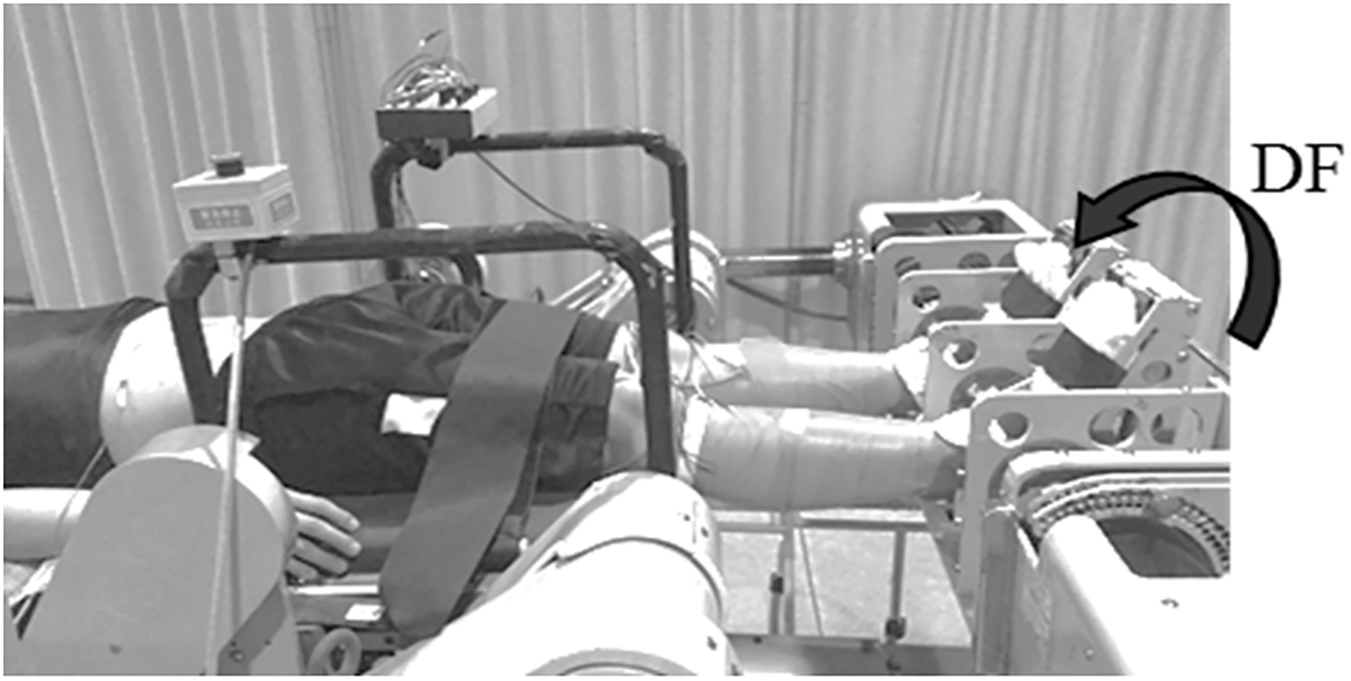
Experimental setup. The machine was utilized to induce muscle stretching in the right calf muscles. The right ankle joint was passively moved into dorsiflexion by the rotation of the foot-plate. The black arrow in this figure indicates the direction of torque applied by the machine. Abbreviation: DF, dorsiflexion.

A specially designed machine [26] was utilized to induce muscle stretching in the right triceps surae muscles with subjects in the supine position (Fig 1), as it is easier to elicit a spinal reflex in the supine position than the prone position [27]. Each foot was strapped to a footplate connected to a servo-controlled motor torque device. The axis of rotation of the ankle joint was adjusted to the axis of rotation of the footplate. Before muscle stretching, the hip, knee, and ankle joints were set to a neutral position (0◦ flexion-/extension). To induce muscle stretching in the right triceps surae muscles, the right ankle joint was passively moved into dorsiflexion by the rotation of the foot-plate. Three dorsiflexion torques of approximately 5, 10, and 15 Nm (stretching intensity; Weak, Medium, and Strong) were applied to stretch the right triceps surae muscles. To prevent the stretch reflex from occurring during each muscle stretching session, the dorsiflexion torques (i.e., at 5, 10, and 15 Nm) were imposed by ramp-and-hold torque trajectories. The duration of the ramp phase was approximately 15 s, and the hold phase was 60 s. Twelve motor responses were evoked in each muscle by the tSCS, as described below, before (Pre), during (During), immediately after (Post 0 min), and 5 min after muscle stretching (Post 5 min). The order of the three stretching conditions (Weak, Medium, and Strong) was randomized across subjects. The time interval between each condition was at least 5 min, and the experiment took approximately 1 h to complete.

### Transcutaneous spinal cord stimulation

To evoke the spinal reflexes in multiple lower-limb muscles, a constant current electrical stimulator was used, with the pulse width set to 1 ms (Digitimer, DS7A, UK). A cathode (50 × 50 mm) was placed on the midline of the skin between the spinous process of the higher lumbar vertebrae, and an anode (100 × 75 mm) was placed over the abdomen [28]. Before the experiment, the optimal site for cathodal electrode placement in the higher lumbar vertebrae was selected based on the location where larger responses were produced in multiple lower-limb muscles (L1/L2: n = 9; L2/L3: n = 1). The electrodes were fixed in place using an elastic bandage to prevent their movement during the experiment. The stimulus intensity was adjusted to evoke spinal reflexes in all recorded muscles, and was kept constant throughout each experiment (54.4 ± 14.0 mA, mean ± SD). Based on previous studies [23–25, 29], paired-pulse stimulation (50 ms interval) was used to confirm whether the response was initiated in afferent fibers and that it was a spinal reflex.

### Data acquisition and analysis

Electromyographic (EMG) activities were recorded from the tibialis anterior (rTA), soleus (rSol), medial gastrocnemius (rMG), vastus medialis (rVM), and biceps femoris (rBF) muscles of the right leg, and the tibialis anterior (lTA), soleus (lSol) and biceps femoris (lBF) muscles of the left leg using bipolar Ag/AgCl surface electrodes (Vitrode F-150S, 18 × 36 mm; Nihon Kohden, Japan). After reducing skin impedance using sandpaper and alcohol, the electrodes were placed over the muscle belly with an interelectrode distance of 20 mm. The EMG signals were amplified (×1000) using a bioamplifer system (MEG-6108; input impedance: >100MΩ, CMRR: >80dB, Nihon Kohden, Japan) and filtered using a band-pass filter between 15 Hz and 3 kHz.

All EMG data were digitized at a sampling rate of 10 kHz with a 16 bit A/D converter controlled by a LabView program that was written by the author (National Instruments). Twelve responses (300 ms with 100 ms pre-stimulus) were recorded during each session (Pre, During, Post 0 min, and Post 5 min). For the size of responses evoked by tSCS in each muscle, we evaluated peak-to-peak amplitudes within time windows from 5 to 45 ms after the tSCS. The root mean square of the background EMG was calculated for the period of 50 ms before stimulation. Ankle torque signals were measured by a torque sensor in the motor torque device. The mean torque values were calculated for the period of 50 ms before stimulation. Joint angle signals from the right ankle were measured using an electrogoniometer (SG110, Biometric Ltd, UK). These two signals were sampled at 4 kHz using an A/D converter (PowerLab, AD Instruments, Colorado Springs, CO) and stored on a computer for later analysis.

### Statistical analysis

Data are described as means ± standard error of the mean (SEM). Using paired t-tests, the mean amplitudes of the response to the second stimulus were compared to the amplitude of the first stimulus in each muscle. The mean amplitudes (mV) of the spinal reflex, torque values (Nm), and background EMG activities (μV) were examined using a two-way repeated measured ANOVA (rmANOVA) with Intensity (Weak, Medium, and Strong) and Time (Pre, During, Post 0 min, and Post 5 min) used as factors. When the rmANOVA test showed significant main effects (without interaction), multiple comparisons were performed using the Bonferroni post hoc test. When the rm ANOVA test showed significant interaction effects, simple main effect tests were conducted to examine the source of the significant interactions. Then, each significant simple main effect was followed by the Bonferroni post hoc test. For all tests, P < 0.05 was set as the significance level. For the paired t-test and rmANOVA test, r and η_p_^2^ were respectively calculated as the effect sizes (ES) indices. Statistical analyses were conducted using SPSS software version 11.0 (SPSS, Chicago, IL).

## Results

### Responses elicited by double pulse stimulation

**Fig 2:**
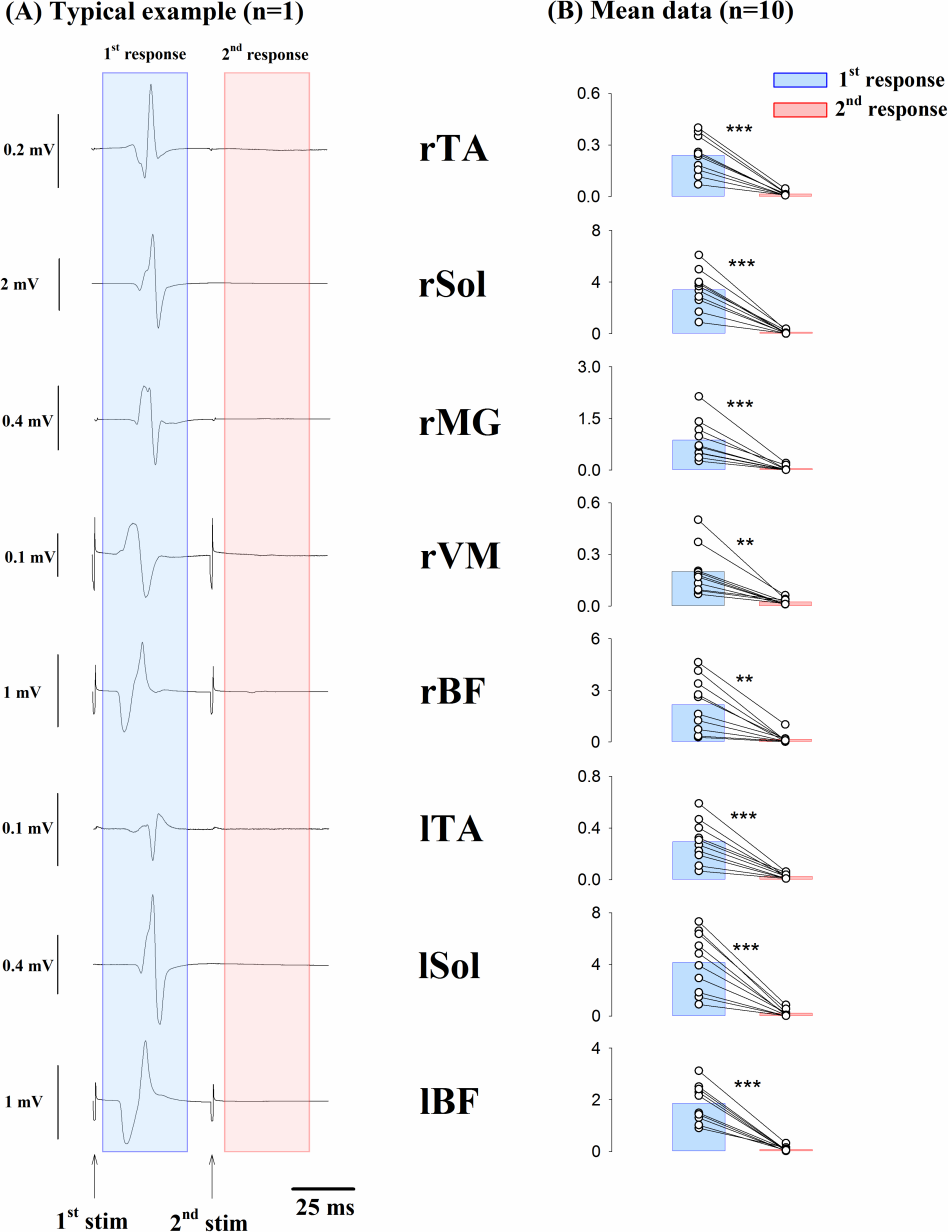
Responses evoked by double pulse stimulation. (A) Representative data from a single subject. Each sweep represents averaged waveform of 12 responses to double pulse stimulation (50-ms time interval) measured in the rTA, rSol, rMG, rVM, rBF, lTA, lSol, and lBF muscles. (B) Averaged group data (n = 10) of peak-to-peak amplitudes (mV) of the responses to the first and second stimulations. Each circle represents an individual data point. Paired t-test revealed significant differences in the group averages between the first and second responses in all of the recorded muscles. **, p<0.01; ***, p<0.001. Abbreviation: rTA, right tibialis anterior; rSOL, right soleus; rMG, right medial gastrocnemius; rVM, right vastus medialis; rBF, right biceps femoris; lTA, left tibialis anterior; lSOL, left soleus; lBF, left biceps femoris.

Fig 2A shows the representative average waveforms obtained from a single subject. The responses to pulses from the second stimulation were noticeably smaller than the responses to the pulses from the first stimulation in all recorded muscles. Fig 2B shows that the mean peak-to-peak amplitude of the second response was significantly smaller than that of the first response in all recorded muscles [rTA: t(9) = 6.990, p = 0.000, ES r = 0.919; rSOL: t(9) = 7.372, p = 0.000, ES r = 0.926; rMG: t(9) = 5.024, p = 0.000, ES r = 0.859; rVM: t(9) = 4.533, p = 0.001, ES r = 0.834; rBF: t(9) = 4.520, p = 0.001, ES r = 0.833; lTA:t(9) = 5.761, p = 0.000, ES r = 0.887; lSOL: t(9) = 5.941, p = 0.000, ES r = 0.893; lBF: t(9) = 8.063, p = 0.000, ES r = 0.937].

### Ankle joint angle and torque

**Fig 3:**
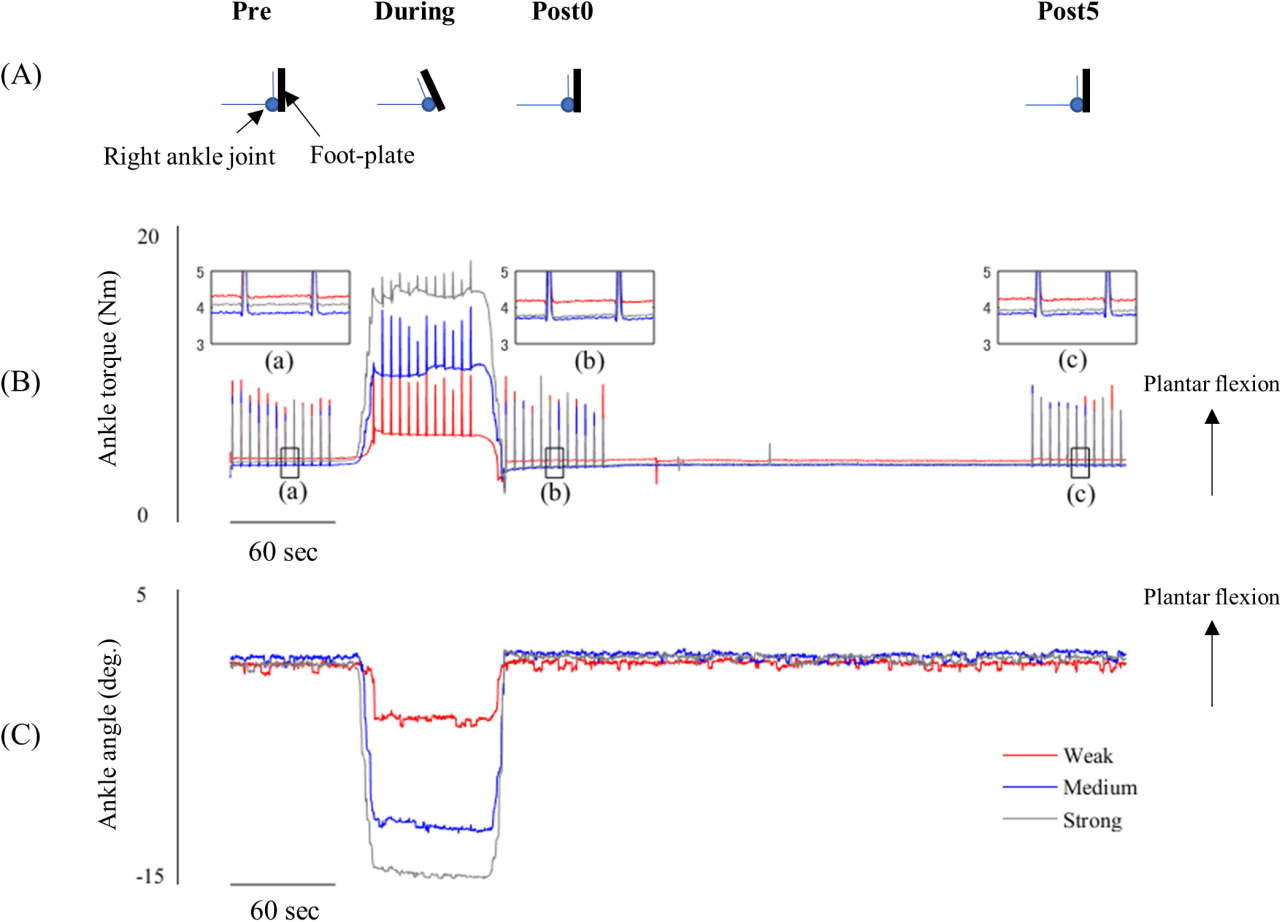
Ankle torque and ankle joint angle. The figure shows a schematic illustration of the movement of the right ankle joint (A) and representative data of ankle torque (B) and ankle joint angle (C) from a single subject. The data of ankle torque (B) and ankle angle (C) that was recorded in each condition were superimposed for comparison (Red line, Weak; Blue line, Medium; Gray line, Strong). Inset figures (a, b and c) respectively indicate zoomed figures of the torque profile before, 0 min and 5 min following stretching in each condition.

Fig 3 shows the changes in the right ankle joint and torque during each condition (Weak, Medium, and Strong). To stretch the right triceps surae muscles, three dorsiflexion torques of approximately 5, 10, and 15 Nm (stretching intensity; Weak, Medium, and Strong) were applied by the motor torque device. When the ankle joint was passively dorsiflexed by the device, a plantar-flexion torque was generated by the viscoelastic properties of muscle-tendon complex of the stretched triceps surae muscles (Fig 3). For the torque data, there were some spikes that were caused by muscle activity of ankle plantar-flexor muscles evoked by tSCS (Fig 3B).

**Fig 4:**
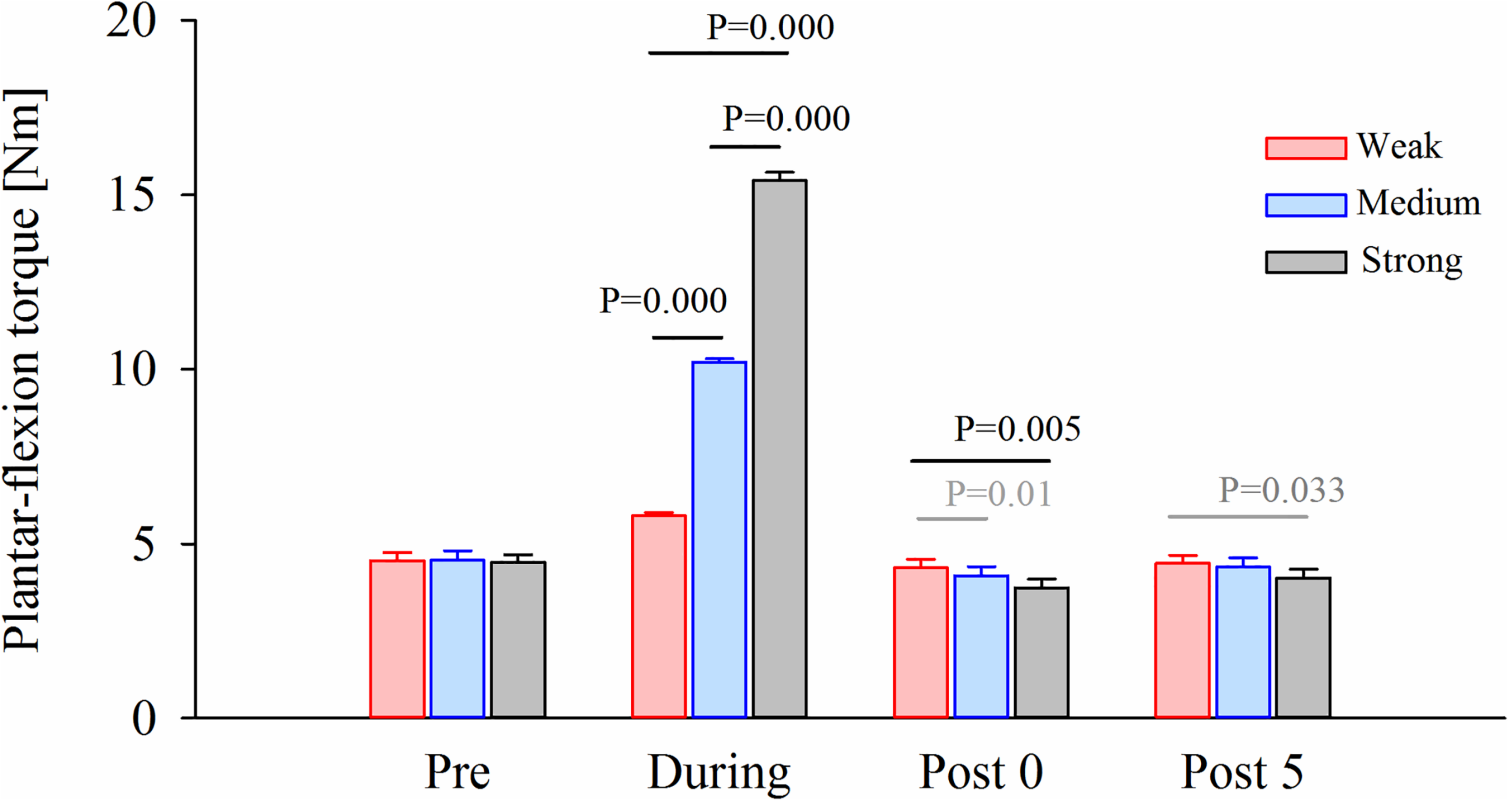
Averaged values of passive-plantar flexion torque produced by the right ankle joint. The averaged values of passive-plantar flexion torque were calculated from the data of 10 subjects. The black lines (p<0.01) and gray lines (p<0.05) indicate the significant difference between intensity conditions.

Fig 4 shows the average torques in each condition. For the average torques, a two-way rmANOVA showed significant main effects for Intensity [F_(2,18)_ = 321.697, p = 0.000, ES η_p_^2^ = 0.973] and Time [F_(1.124,10.114)_ = 990.030, p = 0.000, ES η_p_^2^ = 0.991] and interaction effect [F_(1.221,10.988)_ = 695.161, p = 0.000, ES η_p_^2^ = 0.987]. Since the interaction effect was significant, a one-way rmANOVA was conducted to determine the effect of Intensity (Weak, Medium and Strong) at each time point (Pre, During, Post 0 min and Post 5 min). The main effect of Intensity (Weak, Medium and Strong) was significant During [F_(1.057,9.510)_ = 1037.915, p = 0.000, ES η_p_^2^ = 0.991], Post 0 min [F_(1.305,11.741)_ = 14.334, p = 0.002, ES η_p_^2^ = 0.614], and Post 5 min [F_(2,18)_ = 7.797, p = 0.004, ES η_p_^2^ = 0.464]; however, it was not in the Pre stage [F_(2,18)_ = 0.206, p = 0.815, ES η_p_^2^ = 0.022]. A Bonferroni post-hoc test revealed significant differences of the plantar-flexion torque among the three stretching intensities in the During, Post 0 min, and Post 5 min recordings (Fig 4). Post 0 min, the plantar-flexion torque was 5.1% and 13.2% smaller respectively in the Medium and Strong conditions than in the Weak condition (p = 0.01 and p = 0.005). Post 5 min, the plantar-flexion torque was 9.4% smaller in the Strong condition than in the Weak condition (p = 0.033).

### Changes in the amplitude of spinal reflexes during muscle stretching

**Fig 5:**
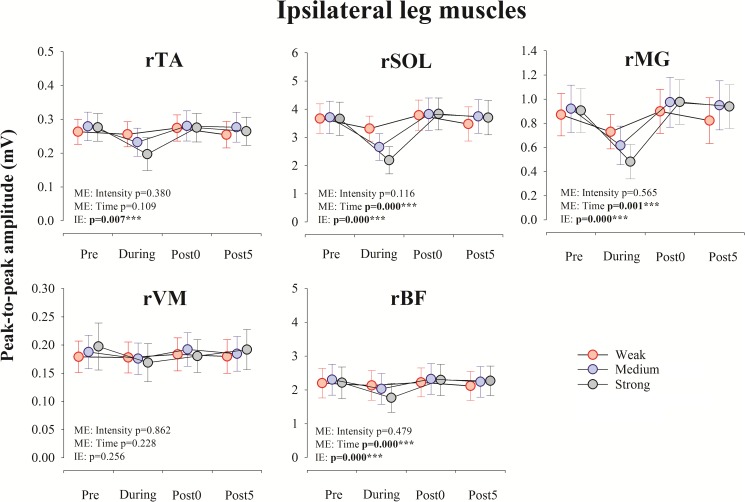
Time course of the amplitude of spinal reflex responses in each muscle of the ipsilateral leg. Averaged amplitude (mV) of the responses in each muscle obtained from the 10 subjects before (Pre), during (During), immediately after (Post 0), and 5 min (Post 5) after muscle stretching. The results of two-way rmANOVA tests are shown in each panel. ***, p<0.001. Abbreviation: ME, main effect; IE, interaction effect; rTA, right tibialis anterior; rSOL, right soleus; rMG, right medial gastrocnemius; rVM, right vastus medialis; rBF, right biceps femoris.

Fig 5 shows the changes in the averaged peak-to-peak amplitude (mV) of spinal reflexes in the ipsilateral leg due to muscle stretching. As described in Fig 5, a two-way rmANOVA showed a significant interaction effect in the averaged amplitudes of the response in rTA, rSOL, rMG, and rBF [rTA: F_(2.013, 18.118)_ = 6.470, p = 0.007, η_p_^2^ = 0.418; rSOL: F_(2.321, 20.887)_ = 16.337, p = 0.000, η_p_^2^ = 0.645; rMG: F_(6, 54)_ = 19.306, p = 0.000, η_p_^2^ = 0.682; rBF: F_(6, 54)_ = 11.181, p = 0.000, η_p_^2^ = 0.554]. Next, simple main effect tests were conducted to examine the source of the significant interactions in these muscles.

**Fig 6:**
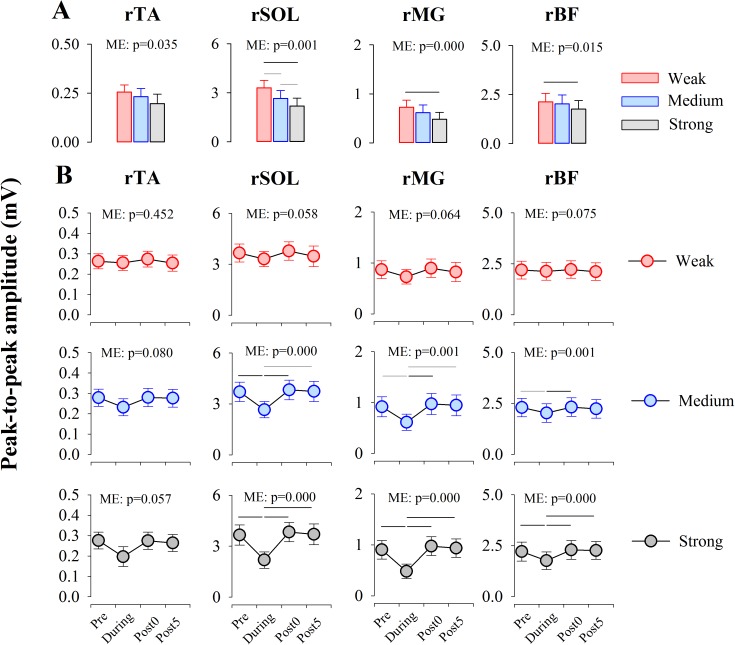
Peak-to-peak amplitude of spinal reflexes in rTA, rSOL, rMG, and rBF. (A) Peak-to-peak amplitudes (mV) of spinal reflexes at each condition (Weak, Medium and Strong) during muscle stretching are shown. A one-way rmANOVA showed the significant main effect (ME) of Intensity in rTA, rSOL, rMG and rBF (the p-values are shown in each panel). (B) Time courses of the amplitude of spinal reflex responses in each muscle of the ipsilateral leg are shown (Red circle, Weak; Blue circle, Medium; Gray circle, Strong). A one-way rmANOVA showed the significant main effect of Time (the p-values are shown in each panel). Black and gray horizontal lines indicate the significant difference between the conditions supported by the post hoc tests (Black, p<0.01; Gray, p<0.05). Abbreviations: ME, main effect; rTA, right tibialis anterior; rSOL, right soleus; rMG, right medial gastrocnemius; rBF, right biceps femoris.

A one-way rmANOVA was conducted to determine the effect of Intensity (Weak, Medium and Strong) at each time point (Pre, During, Post0, and Post5). For the peak-to-peak amplitude (mV) of the spinal reflex in During recording, there were significant main effects for Intensity in rTA, rSOL, rMG, and rBF (rTA: F_(1.292, 11.624)_ = 5.254, p = 0.035, ES η_p_^2^ = 0.369; rSOL: F_(1.210, 10.887)_ = 16.279, p = 0.001, ES η_p_^2^ = 0.644; rMG: F_(2, 18)_ = 15.541, p = 0.000, ES η_p_^2^ = 0.633; rBF: F_(1.301, 11.708)_ = 7.216, p = 0.015, ES η_p_^2^ = 0.445). Bonferroni post hoc tests revealed the significant difference between Intensity conditions in rSOL, rMG, and rBF (the p-values are shown in Fig 6A.).

Then, a one-way rmANOVA was conducted to determine the effect of Time (Pre, During, Post0 and Post5) at each Intensity condition (Weak, Medium and Strong). As described in Fig 6B, there were significant main effects for Time in rSOL, rMG and rBF in Medium (rSOL: F_(1.360, 12.237)_ = 19.625, p = 0.000, ES η_p_^2^ = 0.686; rMG: F_(1.498, 13.482)_ = 14.407, p = 0.001, ES η_p_^2^ = 0.616; rBF: F_(3, 27)_ = 7.374, p = 0.001, ES η_p_^2^ = 0.450) and Strong intensity condition (rSOL: F_(1.252, 11.271)_ = 27.839, p = 0.000, ES η_p_^2^ = 0.756; rMG: F_(1.315, 11.835)_ = 27.266, p = 0.000, ES η_p_^2^ = 0.752; rBF: F_(3, 27)_ = 19.962, p = 0.000, ES η_p_^2^ = 0.689). The Bonferroni post hoc tests reveal that the peak-to-peak amplitudes of the spinal reflexes in rSOL, rMG and rBF in During recording were smaller than those in Pre, Post0, and Post5 recordings (the p-values are shown in Fig 6B.).

**Fig 7:**
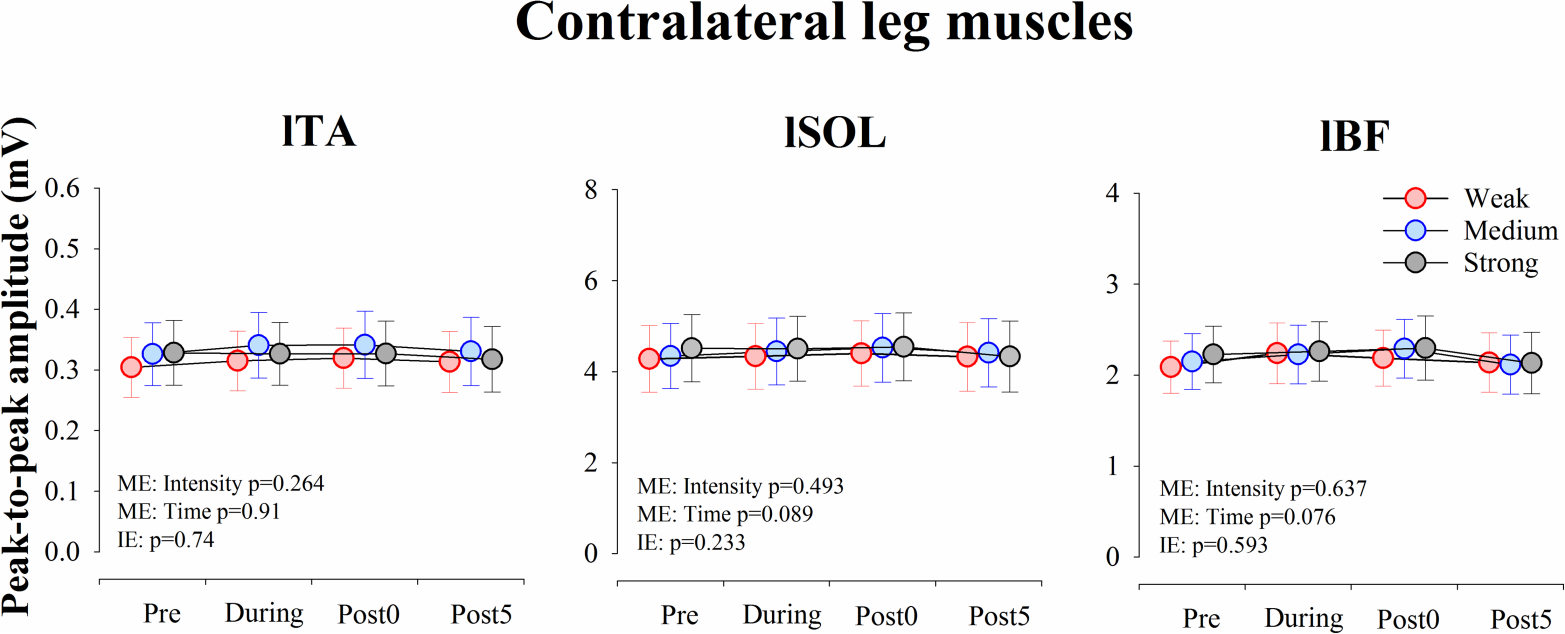
Time course of the amplitude of spinal reflex responses in each muscle of the contralateral leg. Averaged amplitude (mV) of the responses in each muscle obtained from the 10 subjects before (Pre), during (During), immediately after (Post 0), and 5 min (Post 5) after muscle stretching. The results of two-way rmANOVA tests are in each panel. Abbreviations: IE, interaction effect; lBF, left biceps femoris; lSOL, left soleus; lTA, left tibialis anterior; ME, main effect.

On the other hand, as for the averaged peak-to-peak amplitude (mV) of spinal reflexes in the contralateral leg, there were no significant main effects of intensity (lTA: F_(2, 18)_ = 1.433, p = 0.264, ES η_p_^2^ = 0.137; lSOL: F_(2, 18)_ = 0.735, p = 0.493, ES η_p_^2^ = 0.075; lBF: F_(2, 18)_ = 0.463, p = 0.637, ES η_p_^2^ = 0.049) and time (lTA: F_(3, 27)_ = 2.389, p = 0.091, ES η_p_^2^ = 0.210; lSOL: F_(3, 27)_ = 2.411, p = 0.089, ES η_p_^2^ = 0.211; lBF: F_(1.680, 15.122)_ = 3.213, p = 0.076, ES η_p_^2^ = 0.263) and interaction effect (lTA: F_(6, 54)_ = 2.052, p = 0.074, ES η_p_^2^ = 0.184; lSOL: F_(6, 54)_ = 1.396, p = 0.233, ES η_p_^2^ = 0.134; lBF: F_(6, 54)_ = 0.775, p = 0.593, ES η_p_^2^ = 0.079) (Fig 7).

### Background EMG activity

Table 1 shows the values (μV) of background EMG activity in each muscle during the experiment. For background EMG activity in rMG, a two-way rmANOVA showed a significant main effect of Intensity [F_(2,18)_ = 4.282, p = 0.030, ES η_p_^2^ = 0.322], however there was no significant main effect of Time [F_(1.138, 10.241)_ = 1.602, p = 0.238, ES η_p_^2^ = 0.151] and interaction effect [F_(6,54)_ = 0.995, p = 0.404, ES η_p_^2^ = 0.100]. However, the Bonferroni post hoc test did not reveal significant differences between Intensity conditions (P>0.05). No significant main effects of Intensity [rTA: F_(2, 18)_ = 1.496, p = 0.251, ES η_p_^2^ = 0.142; rSOL: F_(2, 18)_ = 0.105, p = 0.901, ES η_p_^2^ = 0.012; rVM: F_(1.032,9.285)_ = 1.051, p = 0.334, ES η_p_^2^ = 0.105; rBF: F_(1.162, 10.462)_ = 0.136, p = 0.757, ES η_p_^2^ = 0.015; lTA: F_(2, 18)_ = 0.522, p = 0.602, ES η_p_^2^ = 0.055; lSOL: F_(1.068, 9.616)_ = 0.627, p = 0.458, ES η_p_^2^ = 0.065; lBF: F_(2,18)_ = 1.208, p = 0.322, ES η_p_^2^ = 0.118], Time [rTA: F_(1.000, 9.001)_ = 1.211, p = 0.300, ES η_p_^2^ = 0.119; rSOL: F_(1.119, 10.071)_ = 0.907, p = 0.375, ES η_p_^2^ = 0.092; rVM: F_(1.185,10.663)_ = 1.986, p = 0.188, ES η_p_^2^ = 0.181; rBF: F_(1.192, 10.729)_ = 0.670, p = 0.457, ES η_p_^2^ = 0.069; lTA: F_(1.564, 14.079)_ = 1.644, p = 0.228, ES η_p_^2^ = 0.154; lSOL: F_(3, 27)_ = 0.439, p = 0.727, ES η_p_^2^ = 0.047; lBF: F_(1.588, 14.295)_ = 1.382, p = 0.277, ES η_p_^2^ = 0.133], and interaction effect [rTA: F_(1.398, 12.581)_ = 1.407, p = 0.271, ES η_p_^2^ = 0.135; rSOL: F_(2.771, 24.943)_ = 2.805, p = 0.064, ES η_p_^2^ = 0.238; rVM: F_(1.509, 13.583)_ = 1.014, p = 0.367, ES η_p_^2^ = 0.101; rBF: F_(1.374, 12.367)_ = 0.624, p = 0.494, ES η_p_^2^ = 0.065; lTA: F_(1.794, 16.146)_ = 0.597, p = 0.545, ES η_p_^2^ = 0.062; lSOL: F_(6, 54)_ = 0.523, p = 0.788, ES η_p_^2^ = 0.055; lBF: F_(2.119, 19.072)_ = 1.730, p = 0.203, ES η_p_^2^ = 0.161] were seen in other muscles.

**Table 1:** Averaged values (μV) of background EMG activities in each muscle obtained from the 10 subjects before (Pre), during (During), immediately after (Post 0), and 5 min after (Post 5) muscle stretching.

		Pre		During		Post 0 min		Post 5 min	
		Mean	SEM	Mean	SEM	Mean	SEM	Mean	SEM
**rTA**	**Weak**	1.23	0.06	4.1	2.74	1.36	0.13	1.33	0.08
	**Medium**	1.3	0.08	5.49	4.07	1.34	0.13	1.28	0.05
	**Strong**	1.35	0.05	6.5	4.3	1.35	0.13	1.29	0.07
**rSOL**	**Weak**	1.14	0.07	1.22	0.16	1.12	0.06	1.32	0.13
	**Medium**	1.14	0.08	1.29	0.2	1.18	0.09	1.19	0.07
	**Strong**	1.14	0.06	1.37	0.22	1.14	0.06	1.13	0.06
**rMG**	**Weak**	1.1	0.07	1.15	0.11	1.12	0.07	1.11	0.08
	**Medium**	1.12	0.07	1.22	0.15	1.09	0.08	1.12	0.07
	**Strong**	1.14	0.07	1.27	0.15	1.13	0.07	1.13	0.07
**rVM**	**Weak**	2.26	0.53	2.25	0.51	2.34	0.54	2.5	0.53
	**Medium**	2.58	0.56	2.28	0.51	2.27	0.54	2.37	0.59
	**Strong**	2.24	0.4	2.18	0.38	2.01	0.28	2.44	0.51
**rBF**	**Weak**	2.09	0.16	2.08	0.16	2.11	0.16	2.07	0.16
	**Medium**	2.03	0.14	2.02	0.16	2.1	0.15	2.08	0.15
	**Strong**	2.07	0.2	2.07	0.19	2.08	0.19	2	0.12
**lTA**	**Weak**	1.55	0.16	1.55	0.15	1.57	0.16	1.59	0.16
	**Medium**	1.67	0.16	1.97	0.43	1.61	0.13	1.58	0.14
	**Strong**	1.81	0.25	1.87	0.2	1.68	0.15	1.58	0.15
**lSOL**	**Weak**	1.56	0.1	1.55	0.09	1.6	0.11	1.58	0.09
	**Medium**	1.66	0.17	1.59	0.11	1.56	0.09	1.58	0.1
	**Strong**	1.78	0.26	1.72	0.25	1.83	0.27	1.84	0.32
**lBF**	**Weak**	1.68	0.2	1.79	0.2	1.85	0.28	1.65	0.2
	**Medium**	1.78	0.23	1.78	0.23	1.89	0.26	2.17	0.43
	**Strong**	1.68	0.21	1.64	0.21	1.68	0.23	1.7	0.25

Abbreviations: lBF, left biceps femoris; lSOL, left soleus; lTA, left tibialis anterior; rBF, right biceps femoris; rMG, right medial gastrocnemius; rSOL, right soleus; rTA, right tibialis anterior; rVM, right vastus medialis; SEM, standard error of the mean.

## Discussion

The aim of this study was to determine whether muscle stretching would affect monosynaptic spinal reflexes in non-stretched muscle as well as stretched muscles. Our results showed that the amplitude of tSCS-evoked responses in both stretched muscles (rSol and rMG) and non-stretched muscle (rBF) in the ipsilateral leg was significantly reduced during muscle stretching of the unilateral triceps surae muscles. In addition, the amplitude of these responses during muscle stretching was significantly smaller as the applied dorsiflexion torque increased. On the other hand, the amplitude of the response in the contralateral leg muscles was not affected by muscle stretching of the unilateral triceps surae muscles. It is well known that muscle stretching inhibits monosynaptic spinal reflex excitability in the stretched muscle [3–8]. The present results extend this knowledge, and suggest that muscle stretching has inhibitory effects on the monosynaptic spinal reflex not only in the stretched muscles, but also in the non-stretched muscles of the ipsilateral leg. These results are discussed below.

### Methodological considerations

First, we need to mention the validity of the method. It is possible that the tSCS-evoked responses were caused by activation of the posterior root, anterior root, or mixed sensory-motor fibers [27]. To confirm that the tSCS-evoked response was caused by the activation of the sensory fibers and was a reflex response, the double pulse stimulation tests were usually used in previous studies [23–25, 29]. If the response resulted solely from the activation of motor nerves, the second response would not be suppressed. On the other hand, if the response was caused by the activation of the sensory fibers, the response evoked by second stimulation would be suppressed due to post-activation depression. The present results of double pulse stimulation tests showed that second responses in all the recorded muscles were completely suppressed by the prior stimulation (Fig 2). Therefore, we concluded that the responses evoked by tSCS in the present study were most likely spinal reflexes.

Since the reflex response evoked by the tSCS has similar characteristics to the H-reflex response, it is thought that the reflex response was primarily generated via the activation of the monosynaptic spinal reflex circuit connecting Ia afferents to motoneurons [23]. However, the reflex response evoked by tSCS results from not only homonymous Ia excitation, but also heteronymous Ia excitation, while H-reflex mainly results from homonymous Ia excitation. This is because tSCS activates the large diameter afferent fibers (i.e., Ia fibers) from multiple lower-limb muscles in the posterior root [24]. Therefore, the response evoked by tSCS in the present study reflects the monosynaptic spinal reflex activated by both homonymous and heteronymous Ia afferents.

The monosynaptic spinal reflex response is modulated by the following mechanisms; centrally or peripherally presynaptic inhibition of Ia terminal, post-activation depression, Ia reciprocal inhibition, Ib inhibition, and so on [30]. In this study, we cannot provide any definitive conclusions about the mechanisms responsible for the inhibition in stretched and non-stretched muscles of the ipsilateral leg observed. However, we speculated the inhibitory mechanisms based on previous studies.

### Possible inhibitory mechanisms

#### Stretched muscles of the ipsilateral leg

There is a lot of evidence to show that spinal reflex excitability in the stretched muscle is suppressed during muscle stretching [3–8]. Using tSCS, we also confirmed in this study that spinal reflex excitability in stretched muscles (i.e., rSol and rMG) is strongly suppressed by stretching the triceps surae muscles. One of the possible explanations for this inhibition is that afferent input induced by muscle stretching inhibits spinal reflex responses [7]. In an earlier study, Robinson et al. reported that the H-reflex in Sol muscles was also inhibited by mechanical pressure on the Achilles tendon when the ankle joint was fixed, and the inhibition induced by muscle stretching was not affected by anesthesia of the skin [7]. Based on these results, Robinson et al. thought that the most probable neural sources for inhibition of spinal reflex excitability were intramuscular receptors (i.e., muscle spindle and Golgi tendon organs), rather than cutaneous and joint receptors [7]. Therefore, afferent input from the muscle spindle and Golgi tendon organs would be involved in the observed inhibition during muscle stretching. Specifically, post-activation depression should be considered. It is well known that previous activation of Ia fibers mediating the afferent volley of the H-reflex produces a reflex depression [30]. This depression is called post-activation depression and considered to be caused by the reduced probability of transmitter release at the Ia synapse [31]. With stretching, Ia fibers would increase their firing rate and induce post-activation depression in the stretched muscles (SOL and MG).

In this study, the degree to which spinal reflexes were suppressed in the ipsilateral leg muscles depended on the applied dorsiflexion torque (i.e., the intensity of a stretch force). In a previous study, it was proposed that the neural mechanisms underlying inhibition change depended on the stretching amplitude (i.e., the dorsiflexion angles of the ankle), i.e. while presynaptic mechanisms may primarily be involved in weak stretching, postsynaptic mechanisms may underlie the inhibitory effect of strong stretching [9]. While the present study was not designed to elucidate the neural mechanisms involved, the proportion of contribution may be related to the relationship between the amount of tSCS-evoked response and the degree of stretch in the stretched muscles.

### Non-stretched muscles of the ipsilateral leg

The present results show that muscle stretching inhibits not only the stretched muscle but also the non-stretched antagonist muscle (rTA). Suppression of the response in this muscle might result from reciprocal inhibition evoked by Ia afferents from the stretched muscles via Ia interneurons [32]. The suppression of spinal reflex excitability during muscle stretching was also observed in the ipsilateral proximal muscles (rBF). It has been reported that afferent inputs from the proximal muscles lead to modulation of the spinal reflexes in distal muscles [33, 34]. However, it remains unknown how the afferent inputs from distal muscles affect spinal reflexes in the ipsilateral proximal muscles. The present results demonstrate the inhibitory effects of muscle stretching of distal muscles on proximal muscles. Since the changes in mechanical properties are not thought to occur in the non-stretched muscles, the decreases in the amplitude of the reflex response could be caused by afferent inputs to the spinal reflex pathway via inhibitory interneurons.

Another candidate is post-activation depression of heteronymous Ia connection between the Ia afferent from the stretched muscles and motoneurons innervating non-stretched muscles. Heteronymous Ia connection is the direct projection of muscle spindle Ia afferents to the motoneurons of various leg muscles, including trans-joint connection between the ankle and leg muscles [14]. The tSCS can induce not only homonymous Ia excitation but also heteronymous Ia excitation. If so, while the triceps surae is stretched, the probability of transmitter release at the heteronymous Ia connection between the Ia afferent from the stretched muscles and motoneurons innervating non-stretched muscles would be reduced because of post-activation depression. Consequently, the response elicited by tSCS in non-stretched muscle decreased as much as its depression.

### Contralateral leg muscles

Crossed effects between the right and left legs have been suggested in various previous human studies [21, 35–37]. For example, one-leg passive pedaling has been reported to inhibit the spinal H-reflex in the contralateral lower-limb muscles [35, 36]. It has also been reported that tibial nerve stimulation on one side modulates EMG activities in the contralateral lower limb muscles (called short-latency crossed response) [21, 37]. On the other hand, in the present study, we could not demonstrate any effect of unilateral triceps surae muscle stretching on tSCS-evoked responses in the contralateral muscles.

Our observations are not contradictory to the previous studies of crossed effects because of the following reasons. First, muscle stretching in the present study involved the single ankle joint movement, whereas passive pedaling includes multi-joint movements of the hip, knee, and ankle joints [35, 36]. It is obvious that the movement-elicited afferent discharge was small during muscle stretching compared to that during passive pedaling. In addition, Collins et al. (1993) reported that the amount of contralateral H-reflex suppression increased with increasing velocity of one-leg passive pedaling [36], suggesting that afferent activities from sensory receptors are strongly related to the suppression. Second, the degree of muscle stretching used in the present study was subthreshold for the activation of stretched muscles, whereas the technique that evokes short-latency crossed response requires strong tibial nerve stimulation that is above the motor threshold in the ipsilateral Sol [21, 37]. Therefore, it is reasonable to consider that the amount and intensity of afferents generated by muscle stretching is not sufficient to observe for crossed effects.

### Limitations

Finally, there are several limitations to this study. First, since the number of EMG electrode was limited, we recorded spinal reflexes from only eight muscles. Therefore, the effects on the other muscles might not be the same as for the muscles tested in this study. Further research is needed to confirm the effects on other muscles. Second, since the present study was designed to investigate the acute effects of muscle stretching, it is unknown whether muscle stretching has chronic effects on the spinal reflexes of multiple lower-limb muscles. A previous study has shown that the maximum H-reflex in Sol muscles decreased after muscle stretching training (10 min/session, 5 times a week, 30 sessions) [1]. Based on that study, it is possible that muscle stretching training has chronic effects on multiple lower-limb muscles.

### Clinical implications

In patients with neurological disorders such as stroke and spinal cord injury, abnormal muscle tone (so-called spasticity) is often present due to hyperexcitability of the spinal reflex pathway. Our results suggest that muscle stretching in a single muscle may relieve these symptoms in multiple muscles. Further studies are required to confirm the neural effects of muscle stretching in patients with neurological disorders with spasticity.
